# Editorial: Antimicrobial resistance (AMR) in foodborne pathogens

**DOI:** 10.3389/fpubh.2026.1968460

**Published:** 2026-09-02

**Authors:** Mabel K. Aworh

**Affiliations:** Department of Biological and Forensic Sciences, Fayetteville State University, Fayetteville, NC, United States

**Keywords:** antimicrobial resistance, foodborne, genomics, One Health, zoonosis

Antimicrobial resistance (AMR) extends far beyond clinical settings. Driven by usage across agriculture, aquaculture, and food processing, resistant pathogens and genes continuously circulate through interconnected animal, environmental, and human ecosystems. This Research Topic brings together four contributions that collectively examine these interconnected dimensions of AMR from animal, environment, food, and human health perspectives. Rather than viewing resistance as an endpoint occurring after infection, the contributions highlight the importance of understanding the epidemiological processes that precede human exposure. Together, they reinforce a central One Health principle: the emergence of foodborne AMR is a distributed process that cannot be adequately understood by examining any single sector in isolation.

An important starting point is the recognition that food-producing animals can harbor clinically important resistant bacteria. Anueyiagu et al. examined extended-spectrum β-lactamase (ESBL)-producing coliforms associated with subclinical mastitis among pastorally managed ruminants. The investigators identified ESBL-producing *Escherichia coli, Klebsiella pneumoniae, K. oxytoca, K. aerogenes*, and *Citrobacter freundii*, including isolates carrying *bla*_*TEM*_ and *bla*_*CTX*−*M*_ resistance determinants. These findings illustrate how resistance of clinical importance can be embedded within livestock-associated bacterial populations, including organisms occurring in apparently less severe or subclinical diseases. The study does not establish transmission from ruminants to humans; rather, it highlights a potential reservoir that warrants surveillance within a broader One Health framework. This distinction is important because identifying resistance within an animal reservoir represents the beginning of a transmission-risk assessment.

The contribution by Wong et al. provides the human disease perspective and demonstrates the consequences of this interconnectedness through an analysis of nontyphoidal *Salmonella* associated with multistate backyard poultry-associated salmonellosis outbreaks in the United States. Across 78 outbreaks involving 6,262 isolates with available resistance information, nearly half of the outbreaks exhibited resistance, while 13% were classified as multidrug resistant and 15% exhibited clinically relevant resistance. Resistant infections were also associated with a higher proportion of hospitalization than infections caused by susceptible isolates. These findings are particularly important because backyard poultry represents an interface at which animal contact, household environments, food production, and human exposure converge. The persistence of resistant *Salmonella* within these outbreaks therefore illustrates how animal-associated reservoirs can have direct public health implications.

The *Salmonella* study highlights the value of integrated AMR surveillance using phenotypic testing and whole genome sequencing (WGS)-based prediction. Resistance varied substantially by serotype, underscoring the need to consider resistance profiles, pathogen lineage, clinical relevance, and epidemiological context. Notably, ciprofloxacin nonsusceptibility predominated among resistant *S*. Enteritidis isolates, highlighting important treatment and surveillance implications.

The study of *Vibrio parahaemolyticus* by Wang et al. extends these questions into aquatic food systems. Using WGS alongside multilocus sequence typing, virulence profiling, and ARGs analysis, the authors characterized 261 clinical isolates from China. ST3 was the predominant sequence type, while *tet(34)* and *bla*_*CARB*−22_ were among the frequently detected AMR-genes (ARGs). The concentration of infections during warmer months and the reporting of aquatic products as food exposures emphasize the importance of environmental and food-production contexts in understanding *V. parahaemolyticus* epidemiology. However, the study did not establish genomic linkage between clinical and food isolates. This limitation underscores a broader challenge for One Health surveillance: epidemiological plausibility alone cannot demonstrate transmission. Establishing the direction and significance of transmission requires coordinated sampling and genomic comparison across humans, foods, animals, and environmental sources.

This challenge is particularly evident in the evidence-chain review by Kong and Jiang, which examines antimicrobial use (AMU) and AMR across the aquaculture-environment-food-human continuum. The review synthesizes evidence linking AMU in aquaculture with selection and enrichment of resistant bacteria and ARGs, while also highlighting the contribution of effluents, sediments, personnel, and equipment to environmental dissemination. Of relevance is the potential role of mobile genetic elements in facilitating horizontal transfer of ARGs within and beyond aquaculture environments. The review therefore moves the discussion from individual resistant pathogens toward the ecological processes that allow resistance to persist and disseminate. Its emphasis on antimicrobial stewardship, biosecurity, effluent management, environmental surveillance, and food-chain hygiene illustrates why interventions directed at only one point in the continuum are unlikely to be sufficient.

The four contributions reveal several important features of foodborne AMR. First, resistance is inherently cross-sectoral. The livestock-associated ESBL findings, backyard poultry-associated *Salmonella* outbreaks, and aquaculture evidence all demonstrate that food production systems can function as important ecological settings for the emergence of resistance. Second, environmental compartments should not be treated as passive recipients of contamination. Water, sediments, and production environments can provide conditions that support persistence and potentially facilitate horizontal movement of ARGs. Third, human disease surveillance remains indispensable. Ultimately, the public health significance of foodborne AMR is determined not simply by the presence of resistance in food or animals, but by whether resistant pathogens reach humans, cause disease, and compromise treatment. Finally, genomic surveillance offers an increasingly powerful bridge between these compartments, but its full potential will depend on coordinated sampling strategies, harmonized methodologies, and epidemiological metadata that permit meaningful comparison across sectors.

These observations also expose important gaps. Detection of the same ARG in different reservoirs does not indicate transmission between them, and genetic similarity alone may not establish the direction of transmission. Future studies should move beyond cross-sectional detection toward longitudinal, multisectoral investigations capable of integrating AMU data, phenotypic susceptibility profiles, resistomes, mobile genetic elements, environmental measurements, and detailed epidemiological information. Such approaches will be essential for distinguishing reservoirs from transmission sources and for identifying the points at which interventions are most likely to interrupt AMR spread.

The next phase of One Health AMR research should focus on where resistance occurs, how it spreads, what sustains it, and how transmission can be prevented. This requires integrated human, veterinary, food-safety, agricultural, and environmental surveillance, supported by genomic approaches to trace resistant pathogens and determinants and translate findings into targeted antimicrobial stewardship, food-production, environmental, biosecurity, and infection-prevention strategies.

This Research Topic highlights the complexity of foodborne AMR and the value of a One Health approach across animals, food, water, environments, and people. Addressing AMR requires identifying where resistance emerges and spreads and intervening before transmission occurs. We hope this Research Topic advances cross-sectoral collaboration and translates molecular and epidemiological insights into effective strategies to protect human and animal health and food safety.

